# Synthesis and Electrochemical Performance of ZnSe Electrospinning Nanofibers as an Anode Material for Lithium Ion and Sodium Ion Batteries

**DOI:** 10.3389/fchem.2019.00569

**Published:** 2019-08-14

**Authors:** Peng Zhou, Mingyu Zhang, Liping Wang, Qizhong Huang, Zhean Su, Liewu Li, Xiaodong Wang, Yuhao Li, Chen Zeng, Zhenghao Guo

**Affiliations:** ^1^State Key Laboratory of Powder Metallurgy, Central South University, Changsha, China; ^2^Department of Biological and Environmental Engineering, Changsha University, Changsha, China

**Keywords:** anode material, Li-ion and Na-ion batteries, electrospinning nanofibers, ZnSe, synthesis, electrochemical performance

## Abstract

ZnSe nitrogen-doped carbon composite nanofibers (ZnSe@N-CNFs) were derived as anode materials from selenization of electrospinning nanofibers. Electron microscopy shows that ZnSe nanoparticles are distributed in electrospinning nanofibers after selenization. Electrochemistry tests were carried out and the results show the one-dimensional carbon composite nanofibers reveal a great structural stability and electrochemistry performance by the enhanced synergistic effect with ZnSe. Even at a current density of 2 A g^−1^, the as-prepared electrodes can still reach up to 701.7 mA h g^−1^ after 600 cycles in lithium-ion batteries and 368.9 mA h g^−1^ after 200 cycles in sodium-ion batteries, respectively. ZnSe@N-CNFs with long cycle life and high capacity at high current density implies its promising future for the next generation application of energy storage.

## Introduction

Over the latest 20 years, lithium-ion batteries (LIBs) have experienced great development to meet the demand of portable electronic devices and hybrid electric vehicles (Mai et al., 2010; Ji et al., 2012; Li W. et al., 2016; Wu F. et al., 2017; Tian et al., 2018). However, the theoretical capacity (372 mA h g^−1^) of the conventional graphite anode in LIBs can't meet the increasing expectations (Li L. et al., 2016; Zhang Y.-C. et al., 2016; Lee et al., 2018). At the same time, Sodium-ion batteries (SIBs), as one of the most competitive alternatives of LIBs, are drawing much attention due to the low cost and high abundance of sodium in the crust (Li et al., 2017; Zhang et al., 2017; Wu et al., 2018). Unfortunately, the larger ionic diameter of Na^+^ (0.106 nm) compared with Li^+^ (0.076 nm) results in the kinetic limitation and larger volume expansion of anode materials, which further leads to reduced capacity in SIBs (Xu et al., 2015; Deng et al., 2018). Therefore, it is a topmost priority to develop high-performance anode materials for LIBs and SIBs (Nitta et al., 2015; Du et al., 2018; Fan and Xie, 2019). Recently, metallic selenides (SnSe_2_, FeSe_2_, CoSe_2_, etc.) have attracted much attention as anode materials thanks to their high energy density and excellent rate performance (Ko et al., 2016; Park et al., 2016; Zhang L. et al., 2016; Cui et al., 2018). Among them, ZnSe is considered as one of the most promising anode materials because of its impressive performance in both LIBs and SIBs (Cao et al., 2018). Particularly, when ZnSe was used as anode materials in LIBs, the Zn reduction by ZnSe could react with Li^+^ to form LiZn and provide additional capacity (Kwon and Park, 2014; liu et al., 2018). However, the pulverization and amorphization of ZnSe during charge and discharge result in poor cycling stability (Fu et al., 2015).

To overcome the problems mentioned above, constructing ZnSe/carbon hybrid material is considered as one of the most effective way to improve electrochemical performance of electrodes. ZnSe shows great synergistic effect with carbon, which highly improves the capacity of anode in energy storage (Zhang et al., 2015). For example, Chen et al. (2017) reported that ZnSe ND@N-PC by using zeolitic imidazolate framework (ZIF-8) and delivered an outstanding capability to LIBs of 1,134 mA·h·g^−1^ at 0.6 A·g^−1^ after 500 cycles. Tang et al. (2018) synthesized a ZnSe microsphere/multiwalled carbon nanotube composite used as SIBs anode materials, which exhibited a high specific capacity of 382 mA h g^−1^ at 0.5 A g^−1^ after 180 cycles. Cao et al. dispersed ZnSe nanoparticles in reduced graphene oxides to synthesize ZnSe-rGO nanocomposite as an anode material for both LIBs and SIBs. The capacity of ZnSe-rGO in LIBs is 530 mA h g^−1^ at 0.5 A g^−1^ after 100 cycles and that of SIBs is 259.5 mA h g^−1^ at 0.1 A g^−1^ after 50 cycles (Cao et al., 2018). But the reported ZnSe/carbon electrodes are mainly microspheres or irregular nanoparticles (Xu Y. et al., 2016). The composite mode and microstructure of ZnSe and carbon are inefficient and then require further design. The specific capacity and stability of the ZnSe/carbon hybrid anode, especially at high current density, also need to be improved (Wang et al., 2017).

In this work, we have successfully synthesized ZnSe nitrogen-doped carbon composite nanofibers (ZnSe@N-CNFs) for both LIBs and SIBs via electrospinning and a simple selenization treatment. This unique one-dimensional (1D) nanostructure has a shorter ion diffusion path and higher electronic conductivity. In the meanwhile, the synergistic effect of encapsulated ZnSe nanoparticles in nitrogen-doped carbon nanofibers can effectively suppress the pulverization and amorphization. As a result, the as-prepared ZnSe@N-CNFs electrodes exhibit an excellent electrochemical performance as anode material for both LIBs and SIBs. The specific capacity of ZnSe@N-CNFs reach to 1,226.1 and 455.0 mA h g^−1^ in LIBs and SIBs, respectively. Meanwhile, the ZnSe@N-CNFs also shows outstanding specific capacity and stability (701.7 mA h g^−1^ after 600 cycles in LIBs and 365.6 mA h g^−1^ after 200 cycles in SIBs) at high current density of 2 A g^−1^. It is expected that the ZnSe@N-CNFs with such great electrochemical performance have promising applications as anodes for both LIBs and SIBs, and would be a direction for design of the other anode materials.

## Experimental Section

### Preparation of ZnSe@N-CNFs

0.5268 g of Zinc acetate dihydrate (C_4_H_6_O_4_Zn·2H_2_O, AR, Sinopharm) was dissolved in 3 ml *N, N*-dimethylformamide (DMF, AR, Sinopharm). 0.4534 g polyacrylonitrile (PAN, Mw 150000, Macklin) was dissolved in 3 ml DMF under magnetic stirring for 30 min at 60°C. The two solutions were mixed and stirred for another 12 h. Then the mixture was transferred into a syringe to electrospin with the distance 20 cm and voltage 13 kV, respectively. The obtained nanofibers were mixed with 1.3 g Selenium powder (99.9%, Aladdin) and calcined at 650°C for 2 h (a ramp rate of 10°C min^−1^) in a tube furnace under vacuum to produce the ZnSe@N-CNFs.

In the meantime, carbon nanofibers (CNF) and ZnSe@N-C were prepared as comparison samples. The carbon nanofibers (CNFs) were synthesized in the same steps without adding Zinc acetate dihydrate. The ZnSe@N-C was synthesized by precursor solution directly dried at 80°C overnight and calcined at the same way with Se powder.

### Materials Characterization

The crystal structure and the composition of the samples were investigated by X-ray diffraction (XRD, Rigaku Dmax/2550VB + 18 kW) and energy dispersive X-ray spectroscopy (EDX, FEI Nova Nano SEM230). The morphology and microstructure of samples was observed through scanning electron microscopy (SEM, FEI Nova Nano SEM230) and transmission electron microscopy (TEM, JEOL JEM-2010). The thermogravimetric analysis (TGA) was tested in air at a ramp rate of 10°C min^−1^. The Raman spectrum test was conducted on a Renishaw in Via 2000. The specific surface areas and the pore size distribution were measured by the Brunauer-Emmett-Teller (BET) test and Barrett-Joyner-Halenda (BJH) method, respectively. The surface chemical composition of the sample was tested by the X-ray photoelectron spectroscopy (XPS, Thermo Scientific ESCALAB 250XI).

### Electrochemical Characterization

A slurry made of ZnSe@N-CNFs (80 wt.%), acetylene black (10 wt.%), and carboxymethyl cellulose sodium (10 wt.%) was dissolved in deionized water and ethanol (3:2), spread onto Cu foil and dried at 80°C for 12 h to prepare the anodes. The LIBs were assembled into 2,032 coin-type cells in an Argon-filled glove box with lithium metal as the reference electrode, LiPF6 (1M) in ethylene carbonate (EC) and dimethyl carbonate (DMC) at a 1:1 volume ratio as electrolyte and polypropylene film (Celgard 2400) as separator. The SIBs were assembled with sodium metal as the reference electrode, NaCF3SO3 (1M) in diethyleneglycol dimethylether (DEGDME) used as electrolyte and glass microfiber (Whatman GF/D) as separator.

All electrochemical tests were carried out at 27°C. The cyclic voltammetry (CV) was measured with voltage window of 0.01–3.0 V by using CHI760E electrochemical workstation. The rate capability and cycle life were tested with LAND CT2001A battery test system. The electrochemical impedance spectroscopy (EIS) tests were carried out with the frequency range of 100 kHz−0.01 Hz by CHI760E electrochemical workstation.

## Results and Discussion

**Figure 1:**
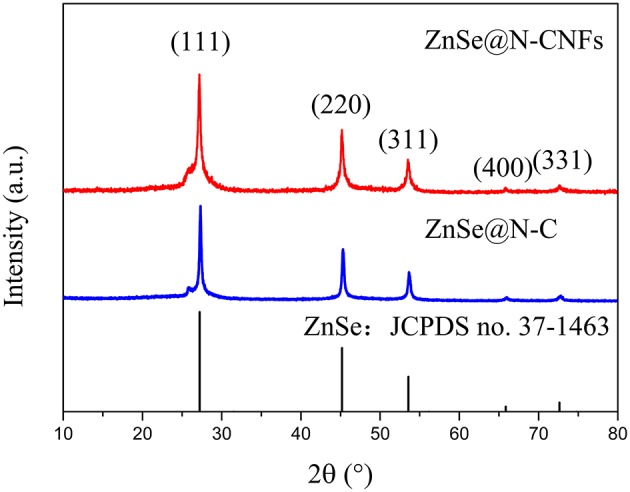
XRD pattern of ZnSe@N-CNFs and ZnSe@N-C.

As shown in XRD pattern of Figure 1 and Figure S1, all the peaks of ZnSe@N-CNFs and ZnSe@N-C can be fully indexed to ZnSe (JCPDS 37-1463), indicate the effective formation of ZnSe as we design (Tang et al., 2018). The wide peak shown at 30° of CNFs is attributed to the amorphous carbon (Lallave et al., 2007). The carbon peaks aren't obvious in the diffraction pattern of ZnSe@N-CNFs and ZnSe@N-C as the highly crystalline ZnSe reflections suppressed those of carbon.

**Figure 2:**
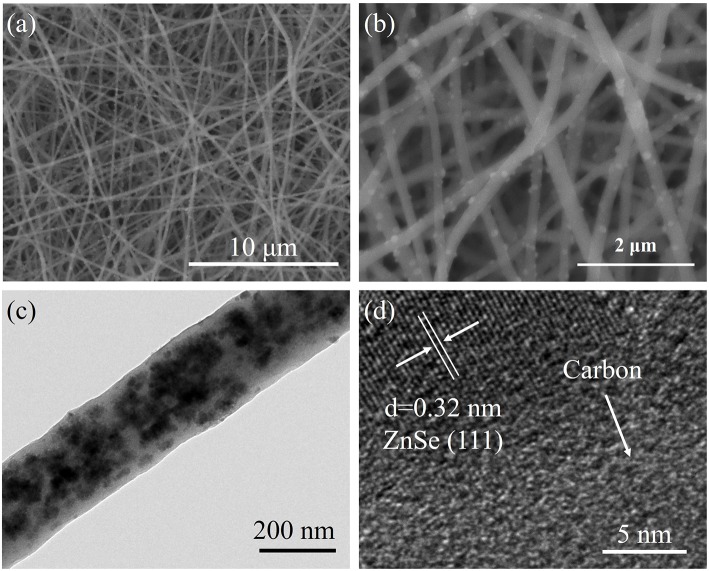
**(a,b)** SEM images of ZnSe@N-CNFs at different magnification. **(c)** TEM image of ZnSe@N-CNFs. **(d)** HRTEM image of ZnSe@N-CNFs.

Figures 2a,b and Figures S2, S3 indicate the SEM image of as-synthesized samples. It can clearly reveal that the ZnSe@N-CNFs and CNFs are continuously fiber structure without aggregated particles. In contrast, ZnSe@N-C is irregular particles. To further reveal the microstructure of ZnSe@N-CNFs, TEM, and HRTEM were used and the images were shown in Figures 2c,d. It is clearly observed in Figure 2c that the morphology of ZnSe@N-CNFs is composed of composite nanofibers of about 200 nm diameter with the ZnSe particles dispersed in the fibers or deposited on their surface. The phenomenon of deposited ZnSe particles on the surface is attributed to the crystal growth during calcined (Ning et al., 2017). Clear lattice fringes can be observed from the HRTEM image in Figure 2d of 0.32 nm corresponding to the (111) plane of ZnSe crystal (JCPDS 37-1463). In addition, the EDX analysis is shown in Figure S4. The atomic ratio of Zn and Se in ZnSe@N-CNFs is about 1:1, matching the stoichiometric ratio of ZnSe compounds. The nitrogen is mainly from PAN, which would increase the conductivity and the number of active sites (Cho et al., 2016).

**Figure 3:**
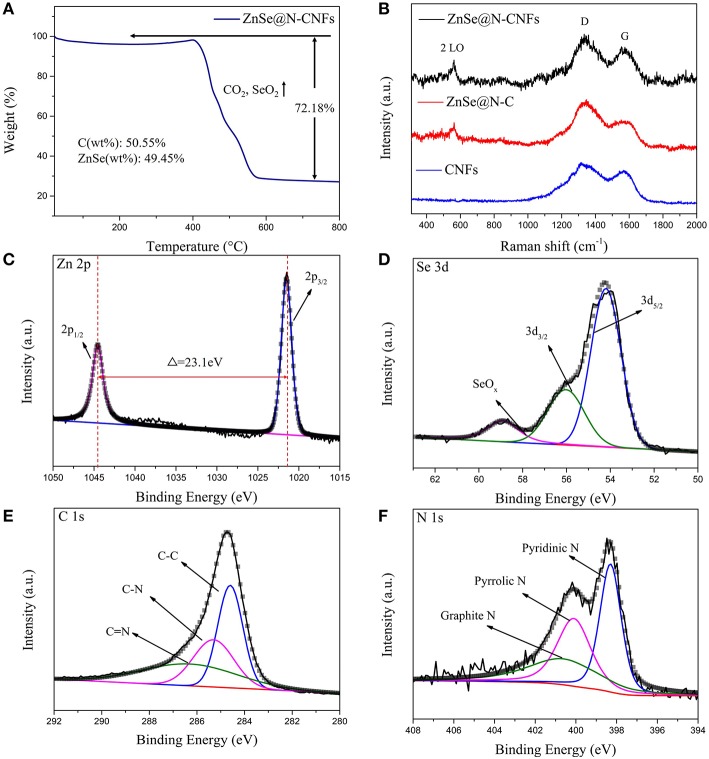
**(A)** TG analysis of ZnSe@N-CNFs. **(B)** Raman spectra of ZnSe@N-CNFs, ZnSe@N-C and CNFs. **(C–F)** XPS spectra of ZnSe@N-CNFs for Zn 2p **(C)**; Se 3d **(D)**; C 1s **(E)**; N 1s **(F)**.

The TGA of ZnSe@N-CNFs was tested from room temperature to 800°C. As shown in Figure 3A, the huge weight loss around 300–600°C correspond to the volatilization of SeO_2_ and CO_2_ (Cui et al., 2017). The mass percentages of ZnSe in ZnSe@N-CNFs is calculated to be 49.45%. In addition, Figure 3B gives the Raman spectra of samples. The two wide peaks can be observed at around 1,340 and 1,586 cm^−1^, which are corresponding with disordered carbon atoms (D band) and graphitic carbon atoms (G band) of carbon, respectively (Wu Q. et al., 2017). The I_D_/I_G_ ratio of the ZnSe@N-CNFs was calculated to be 1.16. The peaks observed at around 500 cm^−1^ in ZnSe@N-CNFs and ZnSe@N-C spectra correspond to 2LO modes of ZnSe (Tang et al., 2018). To further compare the pore size of ZnSe@N-CNFs and ZnSe@N-C, the specific surface was determined by nitrogen adsorption/desorption and the pore size distribution curve calculate by BJH method as shown in Figure S5. The specific surface area of ZnSe@N-CNFs is 30.2 m^2^ g^−1^, higher than 8.1 m^2^ g^−1^ of ZnSe@N-C. The larger specific surface area is attributed to the unique 1D nanostructure. The pore diameter of ZnSe@N-CNFs and ZnSe@N-C are focused on around 3.5–3.8 nm. In addition, the number of pores in ZnSe@N-CNFs is much larger than ZnSe@N-C as shown in Figure S5. Although ZnSe@N-CNFs isn't designed as a porous material, it still shows an excellent specific surface area and suitable mesopore size, which can provide more ion storage active sites and reducing ion migration path (Li et al., 2019).

To investigate the surface physicochemical properties and the chemical composition of ZnSe@N-CNFs, the XPS was measured and the survey spectra of ZnSe@N-CNFs is shown in Figure S6. The peaks corresponding to Zn 2p, Se 3d, C 1s, and N 1s can be clearly observed, which further suggests the presence of Zn, Se, C, and N elements in ZnSe@N-CNFs. As the high resolution of Zn 2p XPS spectrum shown in Figure 3C, two peaks are located at 1,021.5 and 1,044.6 eV, respectively, with an energy difference of 23.1 eV between them, which confirms that the zinc exists as Zn^2+^ form (Ning et al., 2017). The fitted peaks shown in Se 3d spectrum (Figure 3D) at 54.2 and 56.1 eV are corresponding to the Se 3d5/2 and Se 3d3/2 spin orbit, respectively, which indicate the Se mainly exists as Se^2−^. The peak of SeO_x_ locked at 59.0 eV is caused by surface oxidation (Cui et al., 2018). Figure 3D indicate that Se in ZnSe@N-CNFs mainly exists as ZnSe and a small amount of Se has been oxidized to SeO_x_ on the surface. The three fit peaks of high resolution C1s spectrum (Figure 3E) located at binding energy of 284.6, 285.3, and 286.5 eV are related to the C-C bonds, C-N bonds, and C = N bonds, respectively (Liao et al., 2016). Furthermore, the type of nitrogen can be obtained by analyzing the N 1s high-resolution spectrum in the Figure 3F. The fit peaks positioned at 398.3, 400.1, and 400.9 eV can be fit well with pyridinic N, pyrrolic peak N, and graphitic peak N, respectively (Wang et al., 2016).

In view of the special compositional advantages and appealing micro-structures of continuously composite nanofibers ZnSe@N-CNFs as we design as discussed above, these properties should be beneficial for both LIBs and SIBs. The Li-storage and Na-storage properties of samples were further measured to prove the potential of ZnSe@N-CNFs as anodes material.

**Figure 4:**
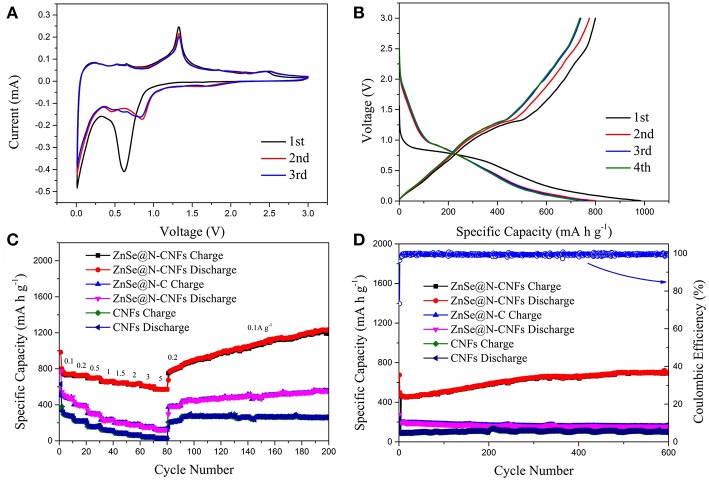
The electrochemical performance of ZnSe@N-CNFs as anodes in LIBs: **(A)** CV curves of the ZnSe@N-CNFs at a scan rate of 0.2 mV s^−1^; **(B)** discharge/charge voltage profiles of the ZnSe/N-CNFs at a current density of 0.1 A g^−1^; **(C)** rate capability at various current densities between 0.1 and 5 A·g^−1^ of the ZnSe@N-CNFs; **(D)** cycling performance and corresponding coulombic efficiency of the ZnSe@N-CNFs at 2 A g^−1^.

Figure 4 shows the electrochemical performance of ZnSe@N-CNFs as anodes in LIBs. The CV measurements were conducted at 0.2 mV s^−1^. As shown in Figure 4A, the peak at 0.6 V in the initial cathodic process is attributed to the reduction from ZnSe to Zn and the formation of a solid electrolyte interface (SEI) layer (Lu et al., 2017). During the follow scan, the curves lapped well, indicating an excellent cycle reversibility. The sharp cathodic peak at around 0.8 V is ascribed to the reduction reaction of Zn^2+^ to Zn^0^. After that, a series of small peaks observed at around 0.5 V are corresponding to the multi-step of forming LiZn alloy (Fu et al., 2016). In addition, the pair of small peaks at 1.6 and 2.3 V are ascribed to the formation and decomposition of Li_2_Se, respectively (Xu Y. et al., 2016). The reaction of ZnSe@N-CNFs anode in LIBs could be described as the following chemical equations (Kwon and Park, 2014):

(1) $$\text{ZnSe}+2{\text{Li}}^{+}+{\text{2e}}^{-}↔\text{Zn }+\;{\text{Li}}_{2}\text{Se}$$

(2) $$\text{Zn }+\;{\text{Li}}^{+}+2{\text{e}}^{-}↔\text{LiZn}$$

Figure 4B shows the discharge and charge profiles of ZnSe@N-CNFs anode at 0.1 A g^−1^ at first four cycles. The voltage window of LIBs is 0.005–3.0 V (vs. Li/Li^+^). For the initial discharge curve, the extended charge plateau at around 0.8 V can be observed, which is due to the decomposition of ZnSe and the formation of the SEI layer. The initial discharge capacity and coulombic efficiency of ZnSe@N-CNFs are 984.7 mA h g^−1^ and 81.3%, respectively. For the next three cycles, the charge/discharge profiles show a similar shape, which the charge/discharge plateau is consistent with the CV curves. The coulombic efficiency improves remarkably from the second cycle, and reaching to 97.6 % for the third cycle and 99.1% for the 4th cycle.

Figure 4C depicts the rate performance of ZnSe@N-CNFs, ZnSe@N-C, and CNFs anode under various current densities from 0.1 to 5 A g^−1^. The ZnSe@N-CNFs anode shows an excellent rate performance of 743.0, 730.3, 699.4, 662.8, 645.8, 636.2, 602.6, and 570.9 mA h g^−1^ at 0.1, 0.2, 0.5, 1, 1.5, 2, 3, 5 A g^−1^, and recovers to 822.7 mA h g^−1^ when the current density reduced back to 0.2 A g^−1^, respectively. In contrast, the capacity of ZnSe@N-C and CNFs anode only has got the values of 43.0 and 26.4 mA h g^−1^ at 5 A g^−1^, and recovers to 379.8 and 228.9 mA h g^−1^ when the current density back to 0.2 A g^−1^. Because of the simple carbonization and without any additional processing, the CNFs exhibits worse Li^+^ storage performance especially at high current density. Furthermore, when the current density is changed to 0.1A g^−1^, the capacity of ZnSe@N-CNFs gradually increases to 1,226.1 mA h g^−1^ after 200 cycles, much higher than ZnSe@N-C and CNFs. The high capacities of ZnSe@N-CNFs is attributed to the special 1D structure. The carbon can effectively prevent the agglomeration of ZnSe particles and lead to more interface of ZnSe and carbon which can provide additional pseudocapacitive capacities In addition, the gradual amorphization process of metallic selenides electrodes during the reversible reactions of anode can provide more interface of ZnSe particle and carbon, which enhance the pseudocapacitive capacities (Gu et al., 2015). More importantly, as shown in Figure 4D, the ZnSe@N-CNFs electrode exhibits excellent discharge capacity of 701.7 mA h g^−1^ after 600 cycles at 2 A g^−1^, and the coulombic efficiency retained over 96% after the first cycle. However, the ZnSe@N-C and CNFs only achieved 155.5 and 99.4 mA h g^−1^ at the same current density after 600 cycles.

**Figure 5:**
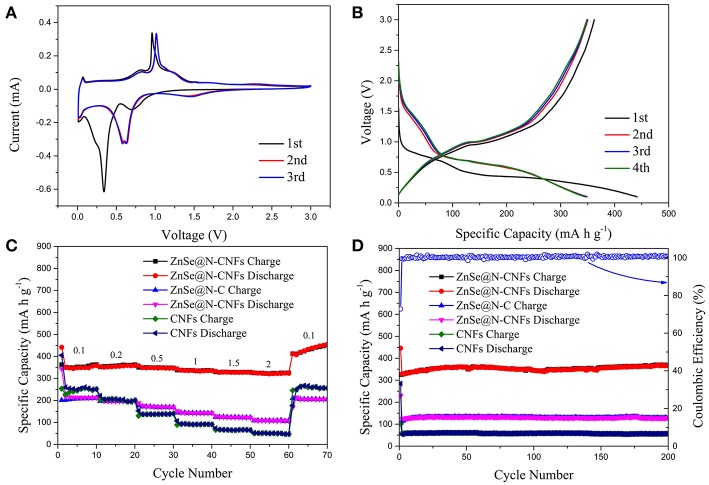
The electrochemical performance of ZnSe@N-CNFs as anodes in SIBs: **(A)** CV curves of the ZnSe@N-CNFs at a scan rate of 0.2 mV s^−1^; **(B)** discharge/charge voltage profiles of the ZnSe/N-CNFs at a current density of 0.1 A g^−1^; **(C)** rate capability at various current densities between 0.1 and 2 A·g^−1^ of the ZnSe@N-CNFs; **(D)** cycling performance and corresponding coulombic efficiency of the ZnSe@N-CNFs at 2 A g^−1^.

Figure 5 displays the electrochemical performance of ZnSe@N-CNFs, ZnSe@N-C, and CNFs as anodes in SIBs. The CV curves of ZnSe@N-CNFs anode in SIBs are shown in Figure 5A at 0.2 mV s^−1^. The obvious peak observed at around 0.4 V in the initial cathodic process is associated with the formation of the SEI layer and insertion of sodium-ion, which is similar to lithium storage (Ge et al., 2015). After that, the following CV curves are well-overlapped, which means the wonderful cycle reversibility of ZnSe@N-CNFs electrodes. The pair of peaks at 0.7 and 1.1 V relate to the transformation of Zn^2+^ and Zn^0^. And the pair of peaks at 1.5 and 2.5 V relates to synthesis and decomposition of Na_2_Se, respectively (Tang et al., 2018). In summary, the reaction of ZnSe@N-CNFs anode in SIBs could be described as following chemical equations (Cao et al., 2018):

(3) $$\text{ZnSe}+2{\text{Na}}^{+}+2{\text{e}}^{-}↔\text{Zn }+\;{\text{Na}}_{2}\text{Se}$$

The cycling performances of ZnSe@N-CNFs at 2 Ag^−1^ with voltage window of 0.005–3 V (vs. Na/Na^+^) were shown in Figure S7. The reversible capacity declined rapidly after 30 cycles. According to previous reports, by increasing the cut-off voltage can reduce the irreversible reactions with carbon and decomposition of electrolytes (Cui et al., 2018). Besides, as Figure 5A shows, there is no obvious reaction peak at low voltage. Therefore, 0.1–3 V (vs. Na/Na^+^) was chosen as the voltage window during discharge and charge test for SIBs. The voltage profiles of ZnSe@N-CNFs with 0.1 Ag^−1^ for the first four cycles are shown in Figure 5B. The first discharge and charge capacities are 441.5 and 362.3 mA h g^−1^, respectively, resulting in a coulombic efficiency of 82.1%. The huge capacity loss is attributed to SEI layers (Park and Kang, 2016). The coulombic efficiency increases rapidly to 100.3% for the second cycle. After the first cycle, the curves coincide well, indicate that the formed SEI layers is very stable and the great cycle reversibility of ZnSe@N-CNFs electrodes in SIBs (Liu et al., 2018).

The rate capability of as-synthesized samples was evaluated at various currents in the range of 0.1–2 A g^−1^, and the results are given in Figure 5C. The capacity of ZnSe@N-CNFs is retained as 358.2, 357.9, 346.1, 334.8, 326.7, and 323.3 mA h g^−1^ at current densities of 0.1, 0.2, 0.5, 1, 1.5, 2 A g^−1^, respectively. After the current densities back to 0.1 A g^−1^, the capacity is raised to 455.0 mA h g^−1^. Moreover, as shown in Figure 5D, the ZnSe@N-CNFs deliver an initial discharge capacity of 446.2 mA h g^−1^ with initial columbic efficiency of 72.9% at 2 A g^−1^. And thereafter from the second cycle on, the columbic efficiency is over 99% and the reversible capacity is 365.6 mA h g^−1^ after 200 cycles. Both the rate capability and cycle performance are much better than ZnSe@N-C and CNFs. Both high capacity and increasing capacity can be attributed to the pseudocapacitive effect and its growth during cycling, the same as LIBs.

**Figure 6:**
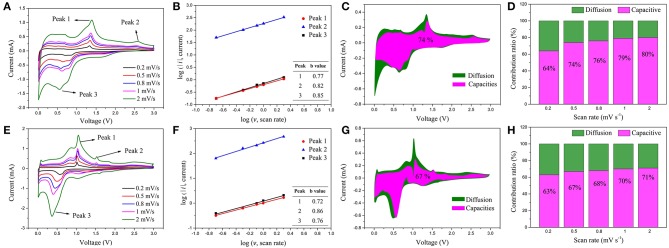
**(A)** CV curves of the ZnSe@N-CNFs electrode for LIBs at different scan rates; **(B)** corresponding log(i) vs. log(v) plots for LIBs at each redox peak (peak current: i, scan rate: v) of the ZnSe@N-CNFs electrode; **(C)** capacitive and diffusion controlled contributions to charge storage of ZnSe@N-CNFs electrode for LIBs at 0.5 mV·s^−1^; **(D)** normalized contribution ratio of capacitive and diffusion-controlled capacities of ZnSe@N-CNFs electrode for LIBs at different scan rates; **(E)** CV curves of the ZnSe@N-CNFs electrode for SIBs at different scan rates; **(F)** corresponding log(i) vs. log(v) plots for SIBs at each redox peak (peak current: i, scan rate: v) of the ZnSe@N-CNFs electrode; **(G)** capacitive and diffusion controlled contributions to charge storage of ZnSe@N-CNFs electrode for SIBs at 0.5 mV·s^−1^; **(H)** normalized contribution ratio of capacitive and diffusion-controlled capacities of ZnSe@N-CNFs electrode for SIBs at different scan rates.

No matter in LIBs or SIBs, the ZnSe@N-CNFs all shows satisfactory performance with high capacity and strong cycling stability. To further investigate the reason for the outstanding performance and evaluate the pseudocapacitive behavior of ZnSe@N-CNFs composite electrodes, the CV tests at different scan rates of 0.2–2 mV s^−1^ in LIBs and SIBs were conducted, as shown in Figures 6A,E. The relation between peak current (*i*) and scan rate (*v*) can describe by following two equations (Xu Y. et al., 2016):

(4) $$i=av^{b}$$

(5) log(i)=blog(v)+loga

When the value of the slope *b* is 0.5, the ion storage process can be considered as diffusion controlled. When the value of slope *b* is 1, the ion storage process can be considered as capacitive controlled (Tang et al., 2018). The value of *b* of ZnSe@N-CNFs anode in LIBs and SIBs is calculated and showing in Figure 6B,F, respectively. The value of *b* in each peak is between 0.5 to 1, which can be considered that the ion storage process of ZnSe@N-CNFs anode is controlled by both capacitive and diffusion (Zhou et al., 2019).

According to the relational equation of *i* = *av* for capacitive processes and *i* = *av* ^1/2^ for diffusion processes, by introducing the adjustable constant parameters as *k*_1_and *k*_2_, the current *i*(*v*) response at fix voltage can be separated to two parts of capacitive and diffusion currents by following equation (Xu D. et al., 2016):

(6) $$i_{\left(v\right)}=k_{1}v+k_{2}v^{1/2}$$

As Figures 6C,G shows, the contribution from capacitive capacity of ZnSe@N-CNFs anode in LIBs and SIBs at 0.5 mV·s^−1^ are calculated to ~74 and 67%, respectively. With the scan rate increase, the contribution of capacitive gradually increases. As the Figure 6D shows, when the scan rate increased to 2 mV·s^−1^, the capacitive contributions of ZnSe@N-CNFs anode in LIBs reach to as high as 80%. And as for SIBs shown in Figure 6H, the capacitive contribution of ZnSe@N-CNFs anode also reaches to as high as 71% at 2 mV·s^−1^.The capacitive contribution for SIBs is lower than that in LIBs, which is attributed to the larger diameter of Na^+^. The large capacitive contribution of ZnSe@N-CNFs indicates that the unique 1D structure can effectively provide the additional capacity, which explains the high capacity in both LIBs and SIBs (Chen et al., 2016). Moreover, as shown in Figure S8, the capacitive contribution increased in both LIBs and SIBs, which proved that the capacity increases along with the cyclic test mainly caused by the capacitive contribution.

**Figure 7:**
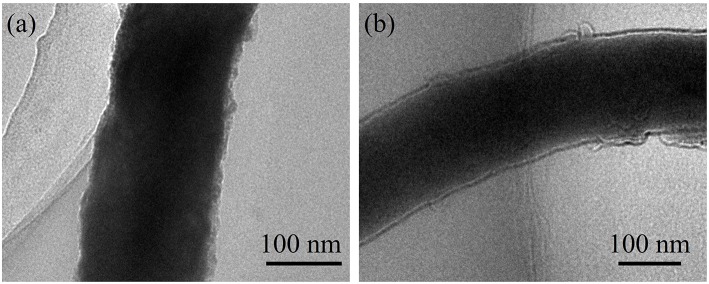
TEM images of ZnSe@N-CNFs composites after 600 cycles at 2 A g^−1^ in LIBs **(a)**; after 200 cycles at 2 A g^−1^ in SIBs **(b)**.

The morphology of ZnSe@N-CNFs anodes after cycles have been observed and shown in Figure 7. Even after long cycles at large current densities, the ZnSe@N-CNFs in both LIBs and SIBs still remain it's 1D structure without additional agglomeration, while the excellent microstructural stability may explain the great cycles stability of ZnSe@N-CNFs. The ZnSe particles disappeared in Figure 7 comparing with Figure 2c after the long cycles is due to the transformation to amorphous phase and, which can provide more interface for the capacitive behavior.

In addition, the EIS analysis was carried out for LIBs and SIBs of ZnSe@N-CNFs to evaluate the conductivity of anode. Figures S9, S10 show the Nyquist plots and fitting curve of ZnSe@N-CNFs anode in LIBs and SIBs, respectively (Cui et al., 2018). The *R*_e_ is the electrolyte resistance, the R_f_ is the SEI layer resistance and *R*_ct_ corresponds to the charge transfer resistance. As the fitting results are shown in Table S1, the R_e_, R_f_ and R_ct_ of the ZnSe@N-CNFs anode in LIBs is 4.74, 1.86, and 8.82 Ω, respectively. It also can be observed that the ZnSe@N-CNFs anode shows great performance of *R*_e_ (9.55 Ω), *R*_f_ (1.95 Ω), and *R*_ct_ (10.37 Ω) in SIBs. The impedance parameters in SIBs is slightly larger than in LIBs, indicating the higher kinetic resistance of SIBs. The satisfactory conductivity performance of ZnSe@N-CNFs anode suggests that ZnSe@N-CNFs can provide a short way for electrons and ions transfer, leading to an outstanding electrochemical performance (Miao et al., 2015; Zhao et al., 2017).

## Conclusion

In summary, the ZnSe@N-CNFs anode was successfully fabricated from 1D electrospinning nanofibers with excellent electrochemical performance in both LIBs and SIBs even at higher current density. The ZnSe@N-CNFs anode delivered a high-capacity of 1,214.0 mA h g^−1^ and 447.5 mA h g^−1^ in LIBs and SIBs, respectively. Furthermore, even the current density was set to 2 A g^−1^, the ZnSe@N-CNFs electrode delivered still maintained at 701.7 mA h g^−1^ after 600 cycles in LIBs and 368.9 mA h g^−1^ after 200 cycles in SIBs, respectively. The remarkable performance is attributed to the high capacitive contribution and stable conductive structure, so that the design scope of the metal selenide electrodes could be further expanded.

## Data Availability

All datasets generated for this study are included in the manuscript/Supplementary Files.

## Author Contributions

MZ supervised the materials synthesis, tests, and manuscript preparation of PZ. All other authors attended part of the work and provided some beneficial advises and discussions on this work.

### Conflict of Interest Statement

The authors declare that the research was conducted in the absence of any commercial or financial relationships that could be construed as a potential conflict of interest.
