# Supplementary material for: Synthesis and Electrochemical Performance of ZnSe Electrospinning Nanofibers as an Anode Material for Lithium Ion and Sodium Ion Batteries
Source: Front Chem. 2019 Aug 14;7:569. doi: 10.3389/fchem.2019.00569 (PMC6702676; doi:10.3389/fchem.2019.00569)
Supplement: Supplementary file 1 [file Data_Sheet_1.docx]

Electronic Supplementary Information (ESI) for Frontiers in Chemistry

**Synthesis and electrochemical performance of** **ZnSe** **electrospinning nanofibers as an anode material for lithium ion and sodium ion batteries**

Peng Zhou^1^, Mingyu Zhang^1^*, Liping Wang^2^, Qizhong Huang^1^, Zhean Su^1^, Liewu Li^1^, Xiaodong Wang^1^, Yuhao Li^1^, Chen Zeng^1^, Zhenghao Guo^1^

^1^ Powder Metallurgy Research Institute, Central South University, Changsha 410083, PR China

^2^Department of Biological and Environmental Engineering, Changsha University, Changsha 410022, PR China

*Corresponding authors. (M. Zhang).

E-mail addresses: zhangmingyu@csu.edu.cn (M. Zhang).





**Fig. S1** XRD pattern of CNFs.


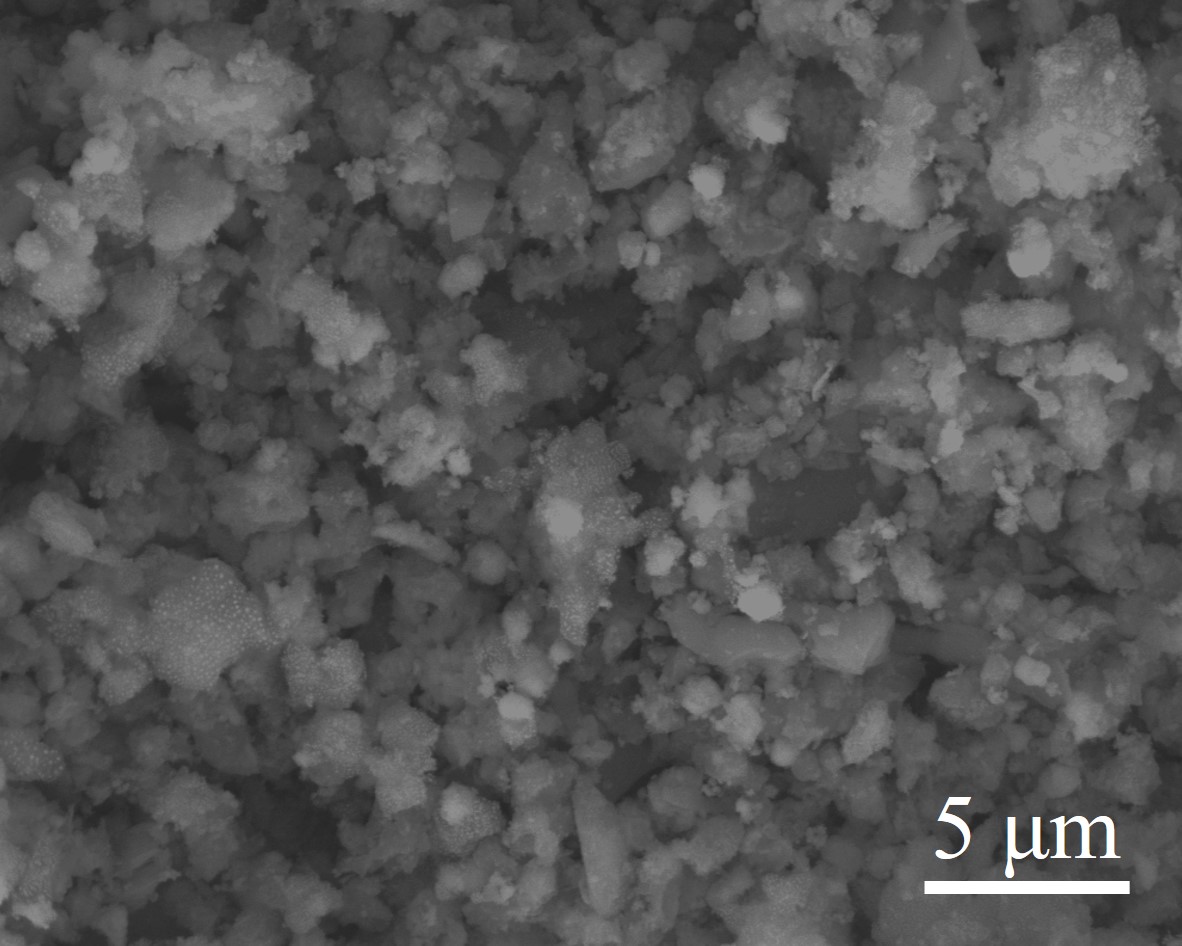


**Fig. S2** SEM of ZnSe@N-C.


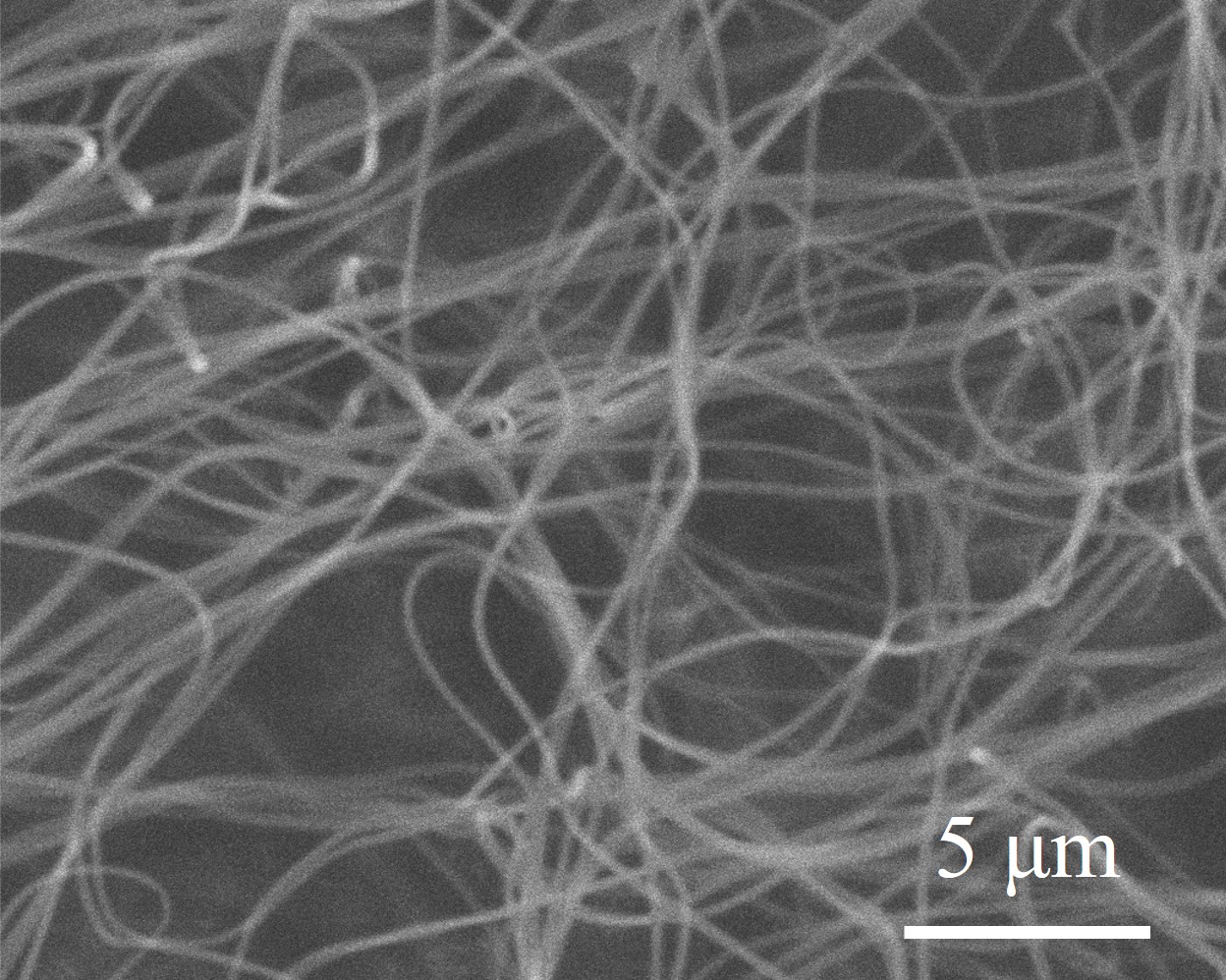


**Fig. S3** SEM of CNFs.


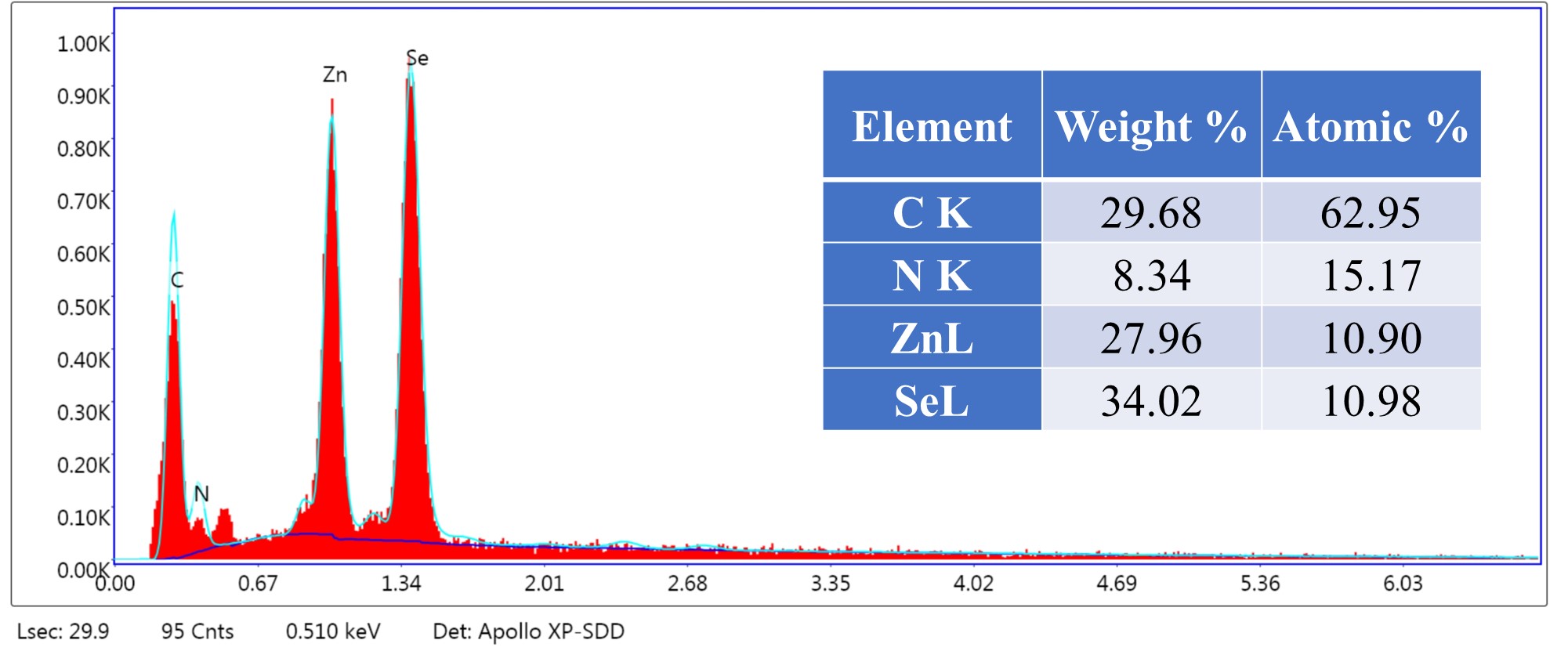


**Fig. S4** EDX spectrum of ZnSe@N-CNFs.


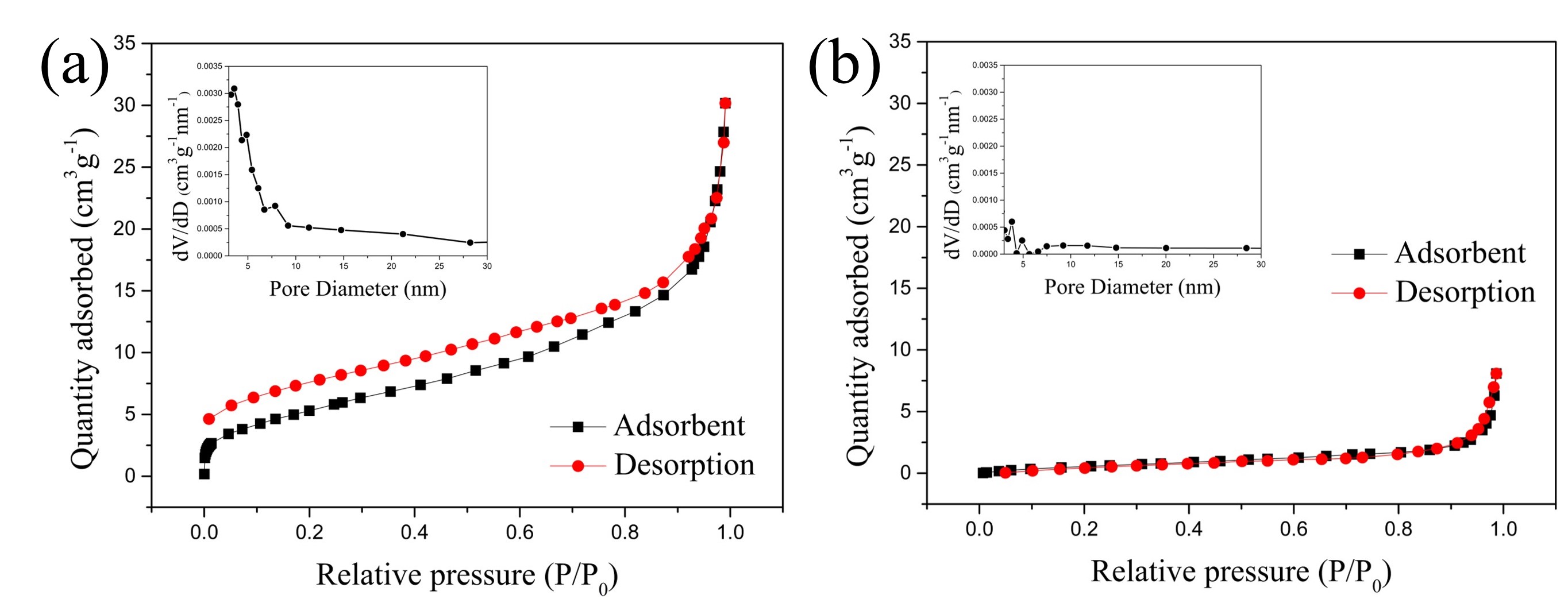


**Fig. S5** N_2_ adsorption isotherms and pore size distribution (insets) of (a) ZnSe@N-CNFs and (b) ZnSe@N-C.





**Fig. S6** XPS survey spectrum of ZnSe@N-CNFs.





**Fig. S7** cycling performance of the ZnSe@N-CNFs with voltage window of 0.005–3 V (vs. Na/Na+) at 2 A g^−1^.


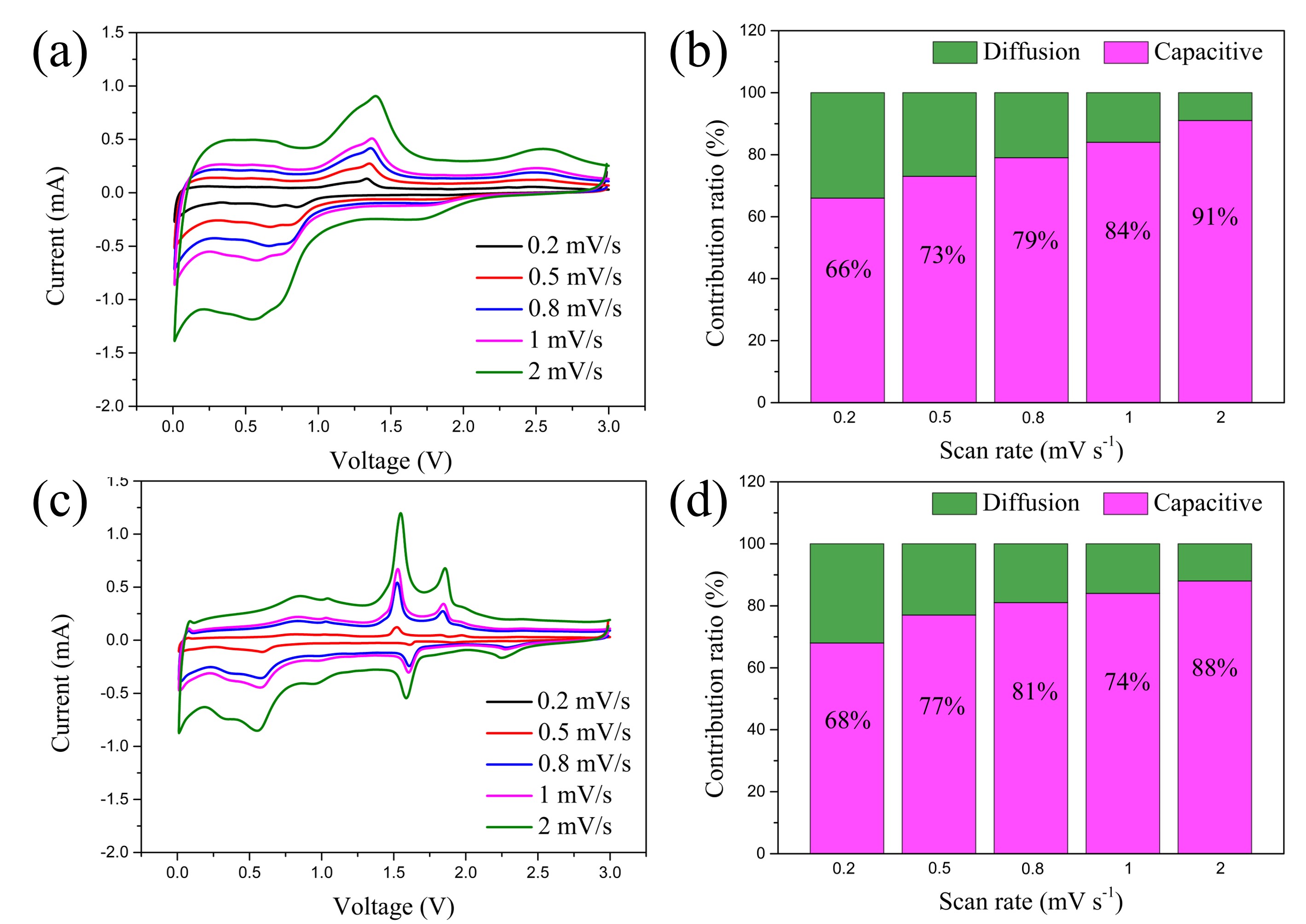


**Fig. S8** (a) CV curves of the ZnSe@N-CNFs electrode for LIBs at different scan rates after 100 cycles; (b) normalized contribution ratio of capacitive and diffusion-controlled capacities of ZnSe@N-CNFs electrode for LIBs at different scan rates after 100 cycles; (c) CV curves of the ZnSe@N-CNFs electrode for SIBs at different scan rates after 100 cycles; (d) normalized contribution ratio of capacitive and diffusion-controlled capacities of ZnSe@N-CNFs electrode for SIBs at different scan rates after 100 cycles.


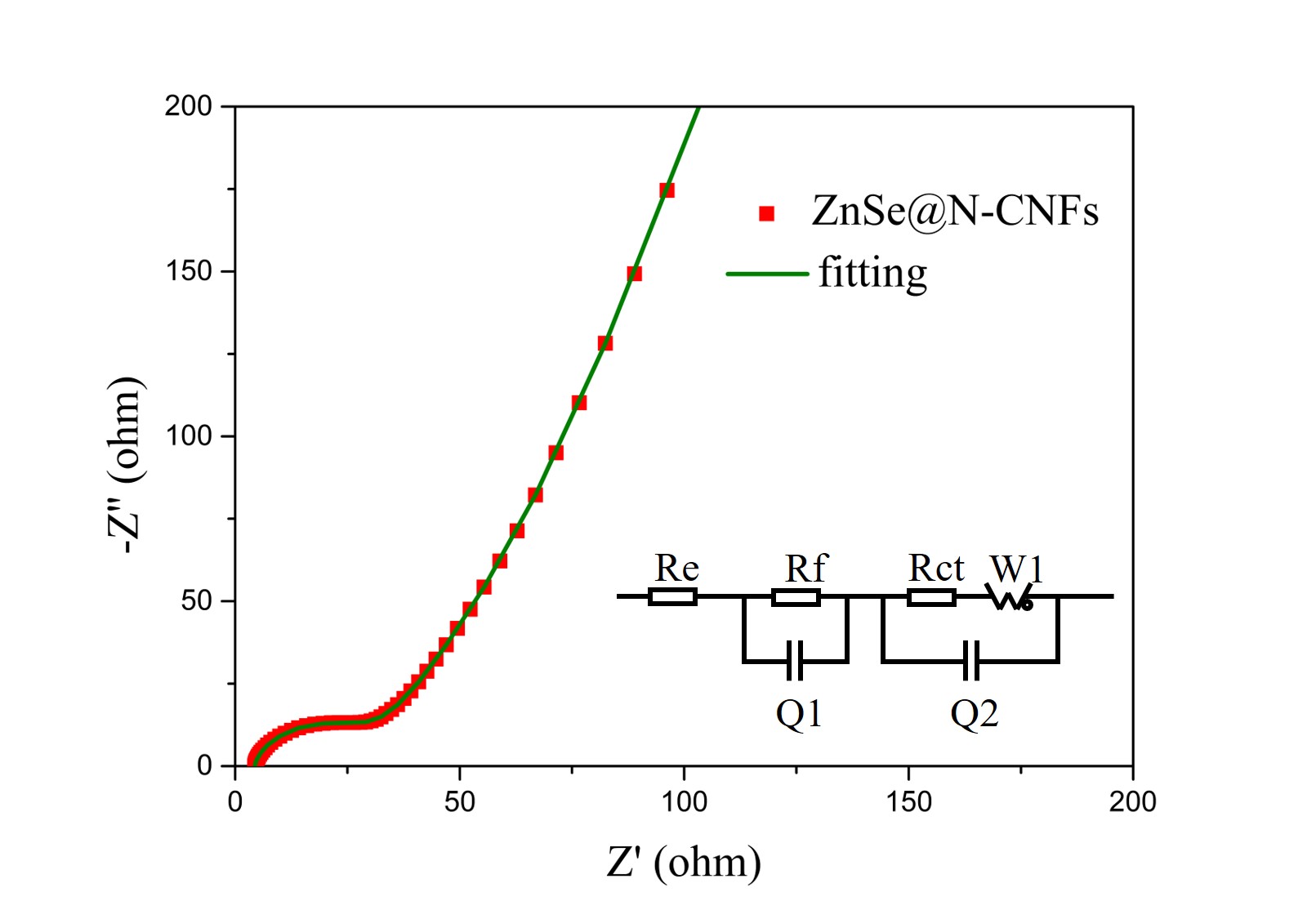


**Fig. S9** Nyquist plots of the ZnSe@N-CNF electrode in LIBs.


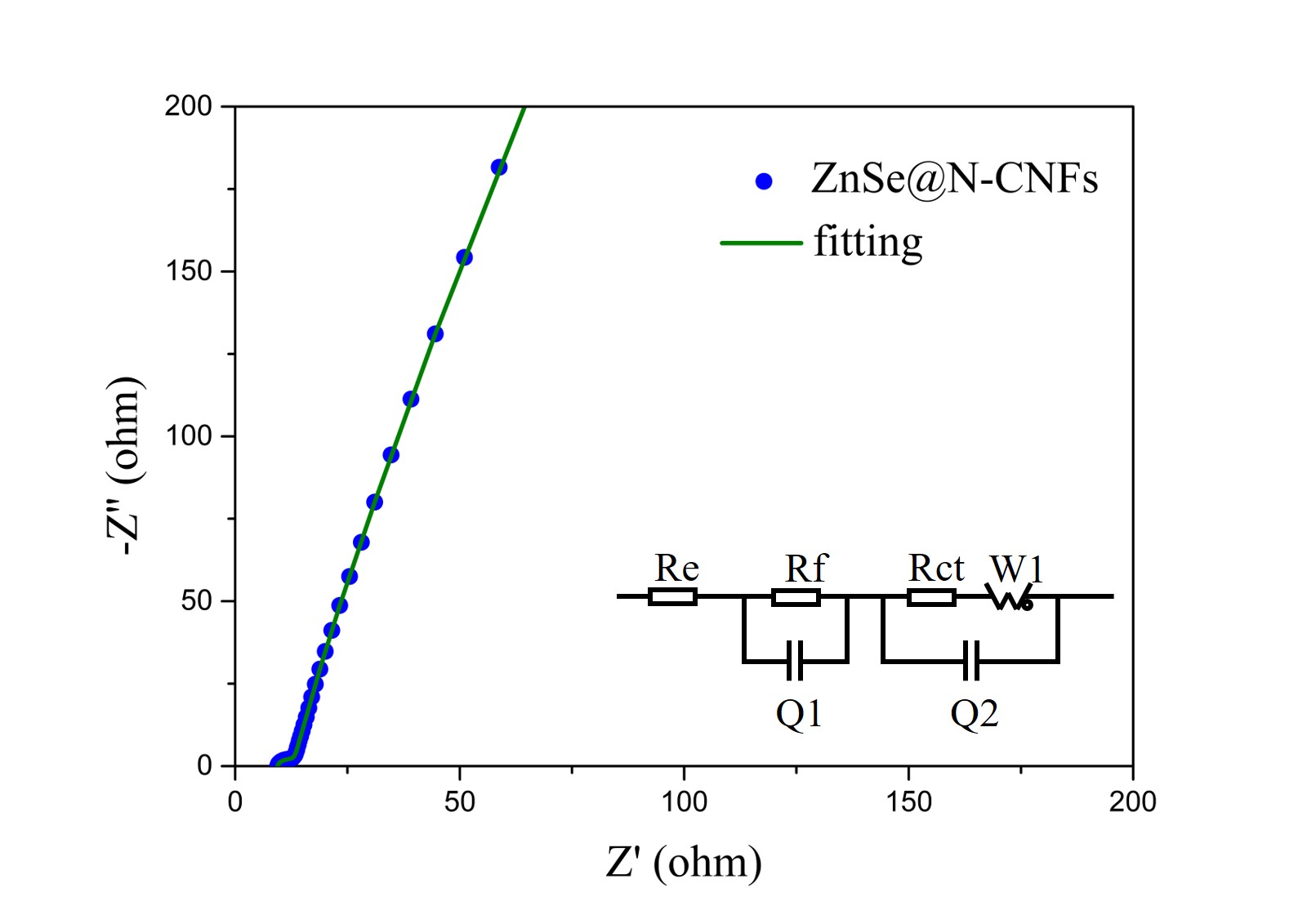


**Fig. S10** Nyquist plots of the ZnSe@N-CNF electrode in SIBs.

**Table S1** Impedance parameters calculated from equivalent circuit.

|  | R_s_(Ω) | R_f_(Ω) | R_ct_(Ω) |
| --- | --- | --- | --- |
| LIBs | 4.74 | 1.86 | 8.82 |
| SIBs | 9.55 | 1.95 | 10.37 |
